# Pattern and Predictors of Maternal Healthcare Services Utilization among Women of Reproductive Age in Lagos, Nigeria

**DOI:** 10.5334/aogh.4570

**Published:** 2025-01-29

**Authors:** Esther Oluwakemi Oluwole, Alero Ann Roberts, Ifeoma Peace Okafor, Victoria Oluwasola Yesufu

**Affiliations:** 1 Department of Community Health & Primary Care, College of Medicine, University of Lagos, Lagos, Nigeria; 2 Unified Initiative for a Drug‑Free Nigeria, Fagba, Lagos, Nigeria

**Keywords:** Pattern, Predictors, Utilization, Maternal Healthcare Services, Antenatal care services, Childbirth services, Postnatal care services, Lagos, Nigeria

## Abstract

*Background:* The world still grapples with alarming maternal mortality rates, particularly in developing nations, including Nigeria. Annual global deaths exceed 500,000, predominantly in developing countries (99%) and sub‑Saharan Africa (over 50%), where the lifetime risk of maternal death is 1 in 26. Millions of women of reproductive age and their children could be saved from poor outcomes through the utilization of available effective affordable maternal healthcare services.

*Objective:* This study assessed the patterns and predictors of maternal healthcare service utilization among women of reproductive age in Lagos state, Nigeria.

*Methods:* A cross‑sectional study was conducted among 453 women of reproductive age selected through multistage sampling between July 2022 and March 2023. Data collection employed interviewer‑administered questionnaires, and analysis was performed using SPSS V.25 software. Statistical analysis included bivariate and multivariate analyses, with a significance level set at *p* < 0.05.

*Findings:* Nearly all participants (99%) were familiar with antenatal care (ANC), while 63% knew about postnatal care services, and 82% understood modern family planning methods. Most respondents (86%) accessed ANC in healthcare facilities; however, the majority (70.7%) booked during the second trimester. The majority (97%) attended ANC more than four times, and 77% gave births in healthcare facilities. Notably, 86% attended postnatal care services primarily for child vaccination. Christian religion (adjusted odds ratio (AOR): 1.810; confidence interval (CI): 0.989–3.313), self‑employment status of spouses (AOR: 2.949: CI: 1.413–6.153), and household monthly income above 60,000.00 naira (AOR: 2.015; CI: 1.002–4.005) were predictors for ANC use. Similarly, Christian religion (AOR: 2.326; CI: 1.426–3.796), self‑employment status of spouses (AOR: 3.111; CI: 1.633–5.929), and having health insurance (AOR: 5.327; CI: 1.229–23.080) were predictors for use of healthcare facilities for childbirth.

*Conclusion:* This study reveals high awareness and utilization of maternal health services but highlights room for improvement in early antenatal care registration and postnatal care beyond the child’s immunization.

## Introduction

Maternal deaths remain a public health challenge in most developing countries, including Nigeria. Maternal health is among the six priority areas in the reproductive health strategy of the country (the social and institutional parameters of women’s health, fertility and family planning, human immunodeficiency virus (HIV)/acquired immune deficiency syndrome (AIDS), reproductive health (RH) of young people, and reproductive organ cancers) to enhance maternal and child health in the continuum of care [1]. The elements of maternal health services include prenatal care, intrapartum care, and postpartum care [2]. Millions of women in the reproductive age group could be saved from poor outcomes through the utilization of available effective affordable maternal healthcare services [3]. The use of maternal health services also contributes to neonatal health outcomes, as the health of the mother and the newborn is closely linked [4].

Global progress in reducing maternal mortality was significant between 2000 and 2020, with a 34% decline. However, stark disparities persist in 2020; nearly 95% of maternal deaths occurred in low‑ and lower‑middle‑income countries. Each day, approximately 800 women lost their lives to preventable pregnancy‑ and childbirth‑related causes, equivalent to one maternal death every 2 minutes [5]. In sub‑Saharan Africa, the lifetime risk of dying from pregnancy is extremely high, and 1 out of every 26 mothers dies as a result of pregnancy and childbirth [5, 6]. Poor or no utilization of maternal healthcare services has been documented as one of the major causes of death among women. The risk of a woman dying from maternal‑related causes during their lifetime in a developing country is about 80 times higher compared with a woman in a developed country [6, 7]. Despite efforts that have been put in place to reinforce maternal healthcare services, maternal mortality is declining at an unacceptably slow pace in most developing countries, including Nigeria. It has remained one of the countries with the highest rates of maternal mortality in the world at 512 deaths per 100,000 live births [2, 8]. About 40,000 maternal deaths occur annually, which account for about 14% of the global total, and the chance of a woman dying from pregnancy and childbirth in Nigeria is 1 in 13 [6, 9]. In addition, for each of the maternal deaths, another 18 women suffer various morbidities, some with long‑term socioeconomic, physical, and psychological consequences [10].

The low level of maternal healthcare‑seeking behavior, including the low proportion of antenatal care use and low proportions of births supervised by skilled birth attendants, are some of the causes of the high prevalence of Maternal Mortality Ratio (MMR) in developing countries [11]. The Sustainable Development Goals have established ambitious health‑related targets for mothers, newborns, and children under the umbrella of Universal Health Coverage by 2030. Addressing the utilization of maternal healthcare services will be fundamental in reducing maternal mortality and achieving the health‑related SDG targets. To this end, the World Health Organization (WHO) has elaborated a global vision where “every pregnant woman receives quality care throughout pregnancy, childbirth and the postnatal period” [9].

Maternal healthcare services, which involve both curative and preventive measures, are crucial for women of reproductive age, and their underutilization, often due to low awareness of rights, inadequate facilities, and unskilled attendants, contributes significantly to high maternal mortality ratio in developing countries. Improving and providing interventions, including access to healthcare facilities, emergency obstetrics and neonatal care services, availability of essential medicines, and skilled workers’ attendance at childbirth among others, is essential. Also, removal of sociocultural, economic, structural, and other barriers has been shown to help reduce maternal mortality [12–15].

The Lagos state government has since constructed mother and child centers (MCC) across the state. The construction of the MCC was one of the outcomes of the Lagos State maternal and child mortality reduction program, which aims to map out strategies for the reduction of the high maternal and child deaths in the state in line with the Sustainable Development Goals (SDGs). The centers provide healthcare services for mothers, babies, and children; a neonatal unit for premature babies; a labor ward with a delivery room; an emergency clinic; and a theater for Cesarean section during complicated deliveries. The program aimed to take maternal and child care close to the people while improving the quality of that care to the highest possible level [16, 17]. This study assessed the pattern and determinants of healthcare facilities utilization for maternal healthcare services among women of reproductive age in Lagos State, Nigeria.

## Methodology

### Description of the study area

Lagos State is in South West Nigeria, with a total land area of 358,862 hectares or 3,577 sq. km. In 2016, an estimate put the population of Lagos State at about 24 million, resulting in a population density of 6,723.5/km [18]. Oshodi Isolo local government area (LGA) is 1 of the 20 LGAs in the state, with 20 wards and an estimated population of 1,000,509 inhabitants, of which 22% are women of childbearing age (WCBA) [19]. The LGA has 82 health facilities, 20 of which are government/public, and a secondary health facility that serves as the referral facility in the LGA. Every ward in the LGA has at least one primary healthcare center (PHC) and several privately owned healthcare facilities.

### Study population

The study population comprised women of reproductive age in the study area.

### Inclusion and exclusion criteria

Only women of reproductive age (15–49 years) who delivered a baby in the past 5 years were interviewed. They were required to have been residents in the study communities for at least 1 year. Those unable to respond to questions appropriately and temporary visitors were excluded.

### Study design and sample size

This was a cross‑sectional study conducted at Oshodi Isolo LGA in Lagos State. The sample size was determined using Cochran’s formula [20], *n* = Z^2^pq/d^2^, using a *p*‑value of 0.67 from Nigeria Demographic and Health Survey (NDHS) 2018 [21], and an acceptable margin of sampling error (d) of 0.05. The minimum calculated sample size was further increased to make up for attrition and improperly filled out questionnaires and to increase precision, and then rounded up to 453.

### Sampling methods

Multistage sampling was used for selecting respondents. In the first stage, 10 wards were selected from the list of wards in the LGA using a simple random sampling technique. In the second stage, 20 streets in each ward were selected by simple random sampling. For the third stage, the streets were divided into quadrants, and a random selection method was employed to establish the initial starting point. A table of random numbers identified the first house and household, and eligible women meeting inclusion criteria were recruited. Subsequent houses and households were selected using systematic sampling, skipping one house and proceeding in a clockwise direction, ensuring comprehensive coverage of all quadrants.

### Data collection methods and tools

Five female community health extension workers (CHEW) were trained and deployed. They possessed excellent communication skills and fluency in English, Yoruba, and Pidgin English, which were the prevalent languages in the study areas. Training encompassed study objectives, questionnaire administration, sampling techniques, informed consent, and data confidentiality.

Data were gathered using a structured questionnaire administered by an interviewer, which was adapted from validated instruments and informed by relevant published research [10, 22]. The data were collected through KoboToolbox, an open‑source tool that facilitates easy gathering and analysis of field data via mobile devices or web forms, both online and offline.

The questionnaire comprised multiple modules, designed to elicit information on sociodemographic characteristics, patterns of maternal healthcare service utilization, and respondents’ experiences during their most recent pregnancy, including antenatal care (ANC), delivery services, postnatal care, and family planning services. Each interview/questionnaire session took between 15 and 20 minutes.

### Monitoring and data quality assurance

To maintain rigor and reliability, a multi‑layered monitoring system was implemented. Field supervisors provided onsite oversight of research assistants, whereas the data manager performed daily online monitoring of collected data. This enabled timely issue resolution and fostered collaborative communication between research assistants and the principal investigator.

### Data management

The data collected were exported in Excel format and subsequently cleaned and analyzed using IBM‑SPSS (now Statistical Product and Service Solutions) version 25. Descriptive statistics were generated, with categorical data presented as proportions and quantitative data summarized as means and standard deviations (SD). To examine relationships between categorical variables, chi‑squared tests were conducted. Binary logistic regression analysis identified predictors of healthcare facility utilization for ANC and childbirth. A significance level of *p* < 0.05 was established as the threshold for statistical significance and variable inclusion in the logistic regression model following bivariate analysis.

## Results

### Sociodemographic/Economic characteristics of respondents and spouses

Table 1 shows that more than half of the respondents (273 (60.3%)) were less than 35 years of age, with a mean age of 33.8 ± 6.1years. Most 436 (96.2%) were married, and 251 (56.5%) got married below or at 25 years of age, and the majority (427 (97.9%)) were in monogamous marriages. Most respondents (434 (95.8%)) and their spouses (444 (98%)) had a secondary level of education or higher. Most (297(65.6%)) were Christian and from the South West region of Nigeria 401 (88.5%). Most respondents (389 (85.9%)) and their spouses (400 (88.3%)) were self‑employed. More than half (288 (65.9%)) earned less than or exactly 60,000.00 naira (₦) monthly, and very few (43 (9.5%)) enrolled in a health insurance scheme.

**Table 1:** Sociodemographic and economic characteristics of respondents.

VARIABLES	FREQUENCY (*N* = 453)	PERCENTAGE (%)
**Age (Years)**		
≤ 35 years	273	60.3
36 years and above	180	39.7
Mean age = 33.81 ± 6.11 years		
**Marital status**		
Not married	17	3.8
Married/cohabiting	436	96.2
**Age at marriage (*n* = 444)**		
≤ 25 years	251	56.5
≥ 26 years	193	43.5
Mean age at marriage = 25.42 ± 3.91 years		
**Type of marriage (*n* = 436)**		
Monogamous	427	97.9
Polygamous	9	2.1
**Level of education of respondents**		
Primary and lower	19	4.2
Secondary and higher	434	95.8
**Level of education of the spouse (*n* = 436)**		
Primary and lower	9	2.0
Secondary and higher	444	98.0
**Religion**		
Islam	156	34.4
Christianity	297	65.6
**Geopolitical zones**		
North	52	11.5
South	401	88.5
**Respondent’s employment status**		
Government/privately employed	64	14.1
Self‑employed	389	85.9
**Spouses’ employment status (*n* = 436)**		
Government/privately employed	53	11.7
Self‑employed	400	88.3
**Household monthly income (**₦**)**		
≤ 60,000.00	288	65.9
> 60,000.00	149	34.1
**Health insurance scheme enrollment**		
No	410	90.5
Yes	43	9.5

### Awareness and utilization of maternal health services during the last childbirth

Table 2 shows that only a few (146 (32.2%)) of the respondents were aware of preconception care services, while almost all (448 (98.9%)) were aware of antenatal care services and 285 (62.9%) were aware of postnatal care services. Only 92 (37.0%) had ever used preconception care services, while the majority (440 (97.1%)) registered for ANC in their last pregnancy, of which 374 (82.5%) registered in healthcare facilities (HCF) and 213 (57.0%) combined both government and private facilities. Of the respondents, 36 (8.0%) registered with a traditional birth attendant (TBA) and 19 (4.2%) with faith homes (refer to maternity facilities associated with religious organizations in Nigeria). Most (311 (70.7%)) registered for ANC in their second trimester (4–6 months gestational age), while 365 (97.6%) received ANC services more than four times, and most (258 (69.0%)) received two doses of tetanus toxoid in the last pregnancy.

**Table 2:** Awareness and utilization of maternal health services during the last childbirth.

VARIABLES	FREQUENCY	PERCENTAGE (%)
**Awareness of maternal health services**		
Preconception care services (*n* = 453)	146	32.2
Antenatal care services (*n* = 453)	448	98.9
Postnatal care services (*n* = 453)	285	62.9
Ever used preconception care services (*n* = 146)	92	63.0
Attended ANC in last pregnancy (*n* = 453)	440	97.1
Used HCF for ANC (*n* = 440)	374	86.0
**Where ANC was received in the last pregnancy (*n* = 374)**		
Government health facilities only	161	43.0
Both (government and private)	213	57.0
**Booking time for ANC in last pregnancy (months) (*n* = 440)**		
1–3	114	25.9
4–6	311	70.7
≥ 7	15	3.4
**Number of ANC visits in the last pregnancy (*n* = 374)**		
≤ 4 times	9	2.4
> 4 times	365	97.6
**Doses of tetanus toxoid received during ANC (*n* = 374)**		
None	7	1.9
One	66	17.6
Two	258	69.0
More than two	43	11.5
Aware of modern family planning services (*n* = 453)	372	82.1
Ever used modern methods of FP (*n* = 372)	166	44.6
*Methods of MFP ever used by respondents (*n* = 166)		
Male condoms	77	46.4
Injectables (Depo Provera)	72	43.4
Implant	66	39.8
IUCD	18	10.8
Combined oral pills	18	10.8
Female condoms	10	6.0
Sterilization	1	0.6
Currently pregnant (*n* = 453)	44	9.7
Planning for pregnancy (*n* = 409)	84	20.5
Currently on modern FP methods (*n*= 325)	127	39.1

IUCD, intrauterine contraceptive device.

*Multiple responses.

The majority (372 (82.1%)) were aware of modern family planning (FP); however, less than half (166 (44.6%)) had ever used modern FP. Male condoms (77 (46.4%)), injectables (Depo Provera) (72 (43.4%)), and implants (66 (39.8%)) were among the methods mentioned. Of the 325 non‑pregnant and non‑intending respondents (79.5%), 127 (39.1%) reported using modern FP methods.

### Utilization of child delivery and postnatal care services during the last childbirth

Table 3 shows that 348 (76.8%) respondents delivered their last baby in HCFs, of which more than half (200 (57.5%)) utilized private HCFs. In almost half of the cases (220 (48.6%)), both the respondents and spouses jointly chose the place of delivery. More than half (188 (54.0%)) of the deliveries were carried out by midwives/nurses, and the majority (386 (85.2%)) had vaginal births. The majority (299 (85.9%)) of the respondents attended postnatal care (PNC) after delivery, and 257 (86.0%) attended because of child immunization at 6 weeks.

**Table 3:** Utilization of child delivery and postnatal care services during the last childbirth.

VARIABLE	FREQUENCY	PERCENTAGE
**Place of childbirth (*n* = 453)**		
Health facility	348	76.8
*TBA	57	12.6
Faith homes	29	6.4
Home/others	19	4.2
**Type of HF used for delivery (*n* = 348)**		
Government HF	148	42.5
Private HF	200	57.5
**Decision of the choice of place of delivery (*n* = 453)**		
Self	87	19.2
Spouse	121	26.7
Both	220	48.6
Others (mother‑in‑law, mother, friends, others)	25	5.4
**Birth attendants (*n* = 348)**		
Doctors	89	25.6
Midwives/nurses	188	54.0
Both	71	20.4
**Mode of child delivery (*n* = 453)**		
Vaginal delivery	386	85.2
Cesarean section	67	14.8
**PNC attendance postpartum (*n* = 348)**	299	85.9
**Time of PNC visit (*n* = 299)**		
Six weeks postpartum	148	49.5
Before 6 weeks postpartum	116	38.8
More than 6 weeks postpartum	35	11.7
*Reasons for PNC attendance (*n* = 299)		
Baby’s immunization	257	86.0
Check‑up	229	76.6
Illness	32	10.7
Family planning	28	9.4

A Traditional Birth Attendant (TBA) is a woman who has gained experience and knowledge in attending births through apprenticeship, self‑study, or traditional practices, but has no formal training as a midwife or other health professional.

*Multiple responses.

### Factors influencing health facility utilization for ANC services

Table 4 shows that respondents’ religion, spouses’ employment status, household monthly income, geopolitical zones, and level of education were the factors associated with the utilization of HCF for ANC (*p* ≤ 0.05).

**Table 4:** Factors influencing health facility utilization for ANC services.

VARIABLE	UTILIZATION OF HF FOR ANC SERVICES				
	NO (*N* = 61; 14.0%)	YES (*N* = 374; 86.0%)	TOTAL (*N* = 435; 100%)	*χ* ^2^	*P*‑VALUE
**Age at marriage**					
≤ 25 years	40 (16.7)	199 (83.3)	239 (100.0)	3.240	0.092
26 years and above	20 (10.6)	168 (89.4)	188 (100.0)		
**Age of respondents (years)**					
≤ 35 years	41 (15.6)	221 (84.4)	262 (100.0)	1.444	0.260
36 years and above	20 (11.6)	153 (88.4)	173 (100.0)		
**Religion**					
Islam	31 (20.9)	117 (79.1)	148 (100.0)	8.917	**0.004**
Christianity	30 (10.5)	257 (89.5)	287 (100.0)		
**Respondent’s employment status**					
Government/private employed	7 (11.7)	53 (88.3)	60 (100.0)	0.321	0.691
Self‑employed	54 (14.4)	321 (85.6)	375 (100.0)		
**Spouses’ employment status**					
Government/privately employed	16 (33.3)	32 (66.7)	48 (100.0)		
Self‑employed	45 (11.6)	342 (91.4)	387 (100.0)	16.687	**< 0.001**
**Monthly income (₦)**					
≤ 60,000.00	46 (16.7)	229 (83.3)	275 (100.0)		
> 60,000.00	12 (8.3)	133 (91.7)	145 (100.0)	5.697	**0.017**
**Geopolitical zones**					
North	12 (25.0)	36 (75.0)	48 (100.0)		
South	49 (12.7)	338 (87.3)	387 (100.0)	5.392	**0.027**
**Respondents’ level of education**					
Primary/no formal	6 (33.3)	12 (66.7)	18 (100.0)		
Secondary and higher	55 (13.2)	362 (86.8)	417 (100.0)	5.807	**0.028**
**Spouses’ level of education**					
Primary/no formal	2 (25.0)	6 (75.0)	8 (100.0)		
Secondary and higher	59 (13.8)	368 (86.2)	427 (100.0)	0.815	0.312*
**Marital status**					
Not married	1(7.1)	13 (92.9)	14 (100.0)		
Married/cohabiting	60 (14.3)	361 (85.7)	421(100.0)	0.568	0.703*
**Health insurance scheme enrollment**					
No	59 (15.0)	334 (85.0)	393 (100.0)		
Yes	2(4.8)	40 (95.2)	42 (100.0)	3.307	0.098*
**Type of marriage**					
Monogamous	60 (14.1)	365 (85.9)	425 (100.0)		
Polygamous	1 (10.0)	9 (90.0)	10 (100.0)	0.137	1.000*

*Fisher’s exact test.

### Factors influencing health facility utilization for childbirth services

Table 5 shows that respondents’ age at marriage, age of respondents, religion, household monthly income, geopolitical zones, level of education, and enrollment in a health insurance scheme were associated with the HCF utilization for childbirth services (*p* ≤ 0.05).

**Table 5:** Factors influencing health facility utilization for childbirth services.

VARIABLE	UTILIZATION OF HF FOR DELIVERY SERVICES				
	NO (*N* = 105; 23.2%)	YES (*N* = 348; 76.8%)	TOTAL (*N* = 453; 100%)	*χ* ^2^	*P*‑VALUE
**Age at marriage**					
≤ 25 years	73 (29.1)	178 (70.9)	251 (100.0)		
26 years and above	30 (15.5)	163 (84.5)	193 (100.0)	11.226	**0.001**
**Age of respondents (years)**					
≤ 35 years	72 (26.4)	201 (73.6)	273 (100.0)		
36 years and above	33 (18.3)	147 (81.7)	180 (100.0)	3.938	**0.053**
**Religion**					
Islam	57 (36.5)	99 (63.5)	156 (100.0)		
Christianity	48 (16.2)	249 (83.8)	297 (100.0)	23.850	**< 0.001**
**Respondents’ employment status**					
Government/privately employed	11 (17.2)	53 (82.8)	64 (100.0)		
Self‑employed	94 (24.2)	295 (75.8)	389 (100.0)	1.502	0.264
**Spouses’ employment status**					
Government/privately employed	25 (47.2)	28 (52.8)	53 (100.0)		
Self‑employed	80 (20.0)	320 (80.0)	400 (100.0)	19.402	**< 0.001**
**Household monthly income (₦)**					
≤ 60,000.00	74 (25.7)	214 (74.3)	288 (100.0)		
>60,000.00	27 (18.1)	122 (81.9)	149 (100.0)	3.170	0.093
**Geopolitical zones**					
North	21 (40.4)	31 (59.6)	52 (100.0)		
South	84 (20.9)	317 (79.1)	401 (100.0)	9.766	**0.003**
**Respondents’ level of education**					
Primary/no formal	9 (47.4)	10 (52.6)	19 (100.0)		
Secondary and higher	96 (22.1)	338 (77.9)	434 (100.0)	6.517	0.022*
**Spouses’ level of education**					
Primary/no formal	4 (44.4)	5 (55.6)	9 (100.0)		
Secondary and higher	101 (22.7)	343(77.3)	444 (100.0)	2.332	0.222*
**Marital status**					
Not married	5 (29.4)	12 (70.6)	17 (100.0)		
Married/cohabiting	100 (22.9)	336 (77.1)	436 (100.0)	0.385	0.559*
**Health insurance scheme enrollment**					
No	103 (25.1)	307 (74.9)	410 (100.0)		
Yes	2 (4.7)	41 (95.3)	43 (100.0)	9.159	**0.004**
**Type of marriage**					
Monogamous	100 (22.7)	341 (75.3)	441 (100.0)		
Polygamous	5 (41.7)	7 (58.3)	12 (100.0)	2.366	0.160*

*Fisher’s exact test.

### Predictors of healthcare facility utilization for ANC and childbirth

Factors with a *p*‑value of ≤ 0.05 from the bivariate analyses were subjected to multivariate logistic regression analysis to obtain the adjusted odds ratios (AORs) and the corresponding 95% confidence interval (CI).

Respondents who were Christian were two times more likely to use healthcare facilities for ANC compared with those who were Muslim. That is, Christian religion had significantly higher odds of ANC use (AOR: 1.810; CI: 0.989–3.313). Also, respondents’ spouses who were self‑employed were three times more likely to use healthcare facilities for ANC compared with those who had government or private employment. That is, self‑employed spouses had significantly higher odds of ANC use (AOR: 2.949; CI: 1.413–6.153). And respondents whose household income per month was above ₦ 60,000.00 were two times more likely to use healthcare facilities for ANC compared with those who earned less. That is, household income above ₦ 60,000.00 per month had significantly higher odds of ANC use (AOR: 2.015; CI: 1.002–4.005).

Similarly, respondents who were Christian were two times more likely to use healthcare facilities for childbirth services compared with those who were Muslim. That is, Christian religion had significantly higher odds of healthcare facility use for delivery (AOR: 2.326; CI: 1.426–3.796). Also, respondents’ spouses who were self‑employed were three times more likely to support the use of healthcare facilities for childbirth compared with those who had government or private employment. That is, self‑employed spouses had significantly higher odds of healthcare facility use for childbirth (AOR: 3.111; CI: 1.633–5.929). And respondents who had health insurance scheme were five times more likely to use healthcare facilities for childbirth compared with those who did not. That is, having a health insurance scheme had significantly higher odds of healthcare facility use for childbirth (AOR: 5.327; CI: 1.229–23.080) (Table 6).

**Table 6:** Predictors of healthcare facility utilization for ANC and childbirth.

VARIABLE	DETERMINANTS OF HEALTHCARE FACILITY UTILIZATION FOR CHILDBIRTH		DETERMINANTS OF HEALTHCARE FACILITY UTILIZATION FOR ANC	
	ADJUSTED ODDS RATIO (95% CI)	*P*‑VALUE	ADJUSTED ODDS RATIO (95% CI)	*P*‑VALUE
**Age of respondents (years)**				
≤ 35 years	*1.0			
36 years and above	1.485 (0.861–2.564)	0.155	—	—
**Age at marriage**				
≤ 25 years	*1.0			
26 years and above	1.524 (0.890–2.610)	0.124	—	—
**Religion**				
Islam	*1.0		*1.0	
Christianity	2.326 (1.426–3.796)	0.001	1.810 (0.989–3.313)	0.054
**Respondents’ level of education**				
Primary/no formal	*1.0		*1.0	
Secondary and higher	1.872 (0.643–5.452)	0.251	2.298 (0.738–7.148)	0.151
**Geopolitical zones**				
North	*1.0		*1.0	
South	2.421 (1.223–4.795)	0.011	2.016 (0.921–4.411)	0.079
**Spouses’ employment**				
Government/privately employed	*1.0		*1.0	
Self‑employed	3.111 (1.633–5.929)	0.001	2.949 (1.413–6.153)	0.004
**Health insurance scheme enrollment**				
No	*1.0		—	—
Yes	5.327 (1.229–23.080)	0.025		
**Household income (₦)**				
≤ 60,000.00			*1.0	
> 60,000.00			2.015 (1.002–4.005)	0.049

*Reference value.

## Discussion

This study found a high level of awareness and utilization of maternal healthcare services for antenatal care and childbirth services among the respondents. The mean age in this study is similar to that of a similar study in South West Nigeria [23]. Most of the respondents were Christian, as in a study in Mekelle City [24]; however, this is in contrast to a Nigerian study where the majority of the respondents were Muslim [25]. Also, many of the respondents were married/cohabiting (96%), and in monogamous family settings (98%). Similar findings have been reported by various studies [26–28]. Respondents (96%) and spouses (98%) showed high education levels, differing significantly from a northern Nigerian study (48.7%) [10]. This reflects regional education disparities, with South West Nigeria exhibiting higher attainment. Only about 10% of the respondents in this study were enrolled in a health insurance scheme, which is consistent with previous research findings that a significant majority in Nigeria lacks health insurance coverage [28–30].

This study found that 32% of the respondents were aware of preconception care service, of which 37% had ever utilized the care. This finding is higher compared with that of a similar study in northern Nigeria that reported 4% awareness and 2.7% utilization [10]. This finding may be because of the variation between South West and northern Nigeria as regards the utilization of healthcare services as documented by NDHS 2018 [21]. Awareness of ANC (99%), PNC (63%), and modern family planning services (82%) was reported in this study, similar to the study in northern Nigeria [10]. This study found that most (82.5%) of the respondents utilized ANC services in healthcare facilities, and almost all (97%) attended ANC services more than four times. This finding is similar to that of a study in Enugu in South East Nigeria, where the majority (90.8%) attended ANC during their last pregnancy [2]. However, these proportions are quite a bit higher compared with the NDHS 2018 report, which states that 67% of women who gave birth in the 5 years preceding the survey received antenatal care from a skilled provider at least once for their last birth. Of women, 57% had four or more ANC visits compared with at least 90% of antenatal visits required for optimal antenatal care [21]. In support of our findings, a comparative community‑based cross‑sectional study in an urban and a rural local government area (LGA) in Lagos, Nigeria, found the majority of the women from the rural (89.3%) and urban (90.3%) areas had at least four ANC visits, and many (84.2% and 83.7%) in rural and urban areas, respectively, attended ANC at a health facility [31]. This study found that 71% of the respondents registered for ANC during the second trimester (4–6 months), while only 114 (26%) did the same during the first trimester. This finding is similar to the study in South West Nigeria, which reported 9.0% of the women attended ANC during the first trimester, while 57.7% attended during the third trimester [23]. This runs contrary to WHO guidelines, which advise initial antenatal care contact before 12 weeks of pregnancy [32]. Also, this study found that 69% of the respondents received at least two doses of tetanus toxoid (TT) injection during their last pregnancy, similar to the NDHS 2018 report, which stated that 62% of women with a birth in the 5 years before the survey received sufficient doses of tetanus toxoid to protect their last birth against neonatal tetanus [21].

Family planning refers to a conscious effort by a couple to limit or space out the number of children they have with contraceptive methods [21]. This study found that 82% of the respondents were aware of modern family planning services; however, only 45% had ever used them, and the current use was 39%. Among the methods ever used by respondents in this study were male condoms (46%), Depo Provera (43%), and implants (40%). This finding is similar to that of a community study in Lagos State, Nigeria, which found 83% of the respondents were aware of family planning services and the prevalence of contraception use was 39%, with the most popular contraceptive method known being the male condom [33]. Also, a comparative study in Imo State, Nigeria, reported 99% and 96% awareness, respectively, and the ever‑used prevalence was 53.9% and 47.2%, while current use was 35.2% and 19.5%, respectively [34]. Similarly, a study in Kano, Nigeria, found an 86.2% level of awareness of modern methods of contraception with a current utilization of 25.6% [35]. We also found about 80% of those not pregnant at the time of data collection for this study were not planning to have a pregnancy; however, only 39% of them were using modern family planning methods. This is similar to the finding of a community study in Ogun State, Nigeria, where more than half (58.1%) of the women were not on contraceptives when they did not intend to get pregnant [36].

Our study revealed that a significant 77% of respondents gave birth in healthcare facilities, surpassing the 2018 NDHS report’s figure of 39% [21] and those of other regional studies in northern (24%) [10] and eastern (50.7%) Nigeria [2]. This notable difference may be attributed to Lagos’ exceptional healthcare system, boasting the highest number of primary healthcare facilities and offering free maternal health services, including delivery, during our data collection period. These findings are corroborated by a comparative community study in Lagos, which reported that 80.3% of rural women and 81.7% of urban women received care from skilled birth attendants [31]. The WHO recommends that, for institutional deliveries, mothers should receive postnatal care within at least 1 day after childbirth. However, if delivery occurs outside a health institution, the first PNC visit should take place immediately or within 1 day of childbirth [37]. This survey found that 86% of the respondents utilized PNC services, of which the majority attended at 6 weeks because of child immunization. A similar study in South East Nigeria found 93% went back to the hospital after 6 weeks post‑delivery [2]. The reported high PNC rates may be inflated, as respondents likely confused PNC visits with routine immunization appointments for their babies at 6 weeks.

This study found statistically significant associations between religion, geopolitical zone, household income, respondents’ level of education, and spouses’ employment status and utilization of HCF for ANC. Respondents from the southern geopolitical zones of Nigeria utilized health facilities for ANC more in their last pregnancy compared with those in the north (*p* < 0.05). This finding corroborates the reports of NDHS that women in the southeast and southwest are more likely to receive antenatal care from a skilled provider and to have four or more ANC visits than women from other zones [21]. Also, studies have reported that women who were better educated had higher incomes and more antenatal care visits than women who were less educated and had lower incomes [38]. Another study on the utilization of maternal health services in Kenya found education and ethnicity to be part of the strong influencers of the first ANC visit [39], while a study in eastern Ethiopia reported that education and religion of women were statistically significantly associated with ANC service utilization [40].

The predictors of HCF utilization for antenatal care services identified among the respondents in this survey include religion, spouses’ employment status, and household income. Similarly, religion, spouses’ employment status, and enrollment in a health insurance scheme were the predictors of HCF utilization for childbirth services.

Men make all the important household decisions and controlled the family’s money in some cultures [41], including ours, and this study found spouses’ employment status to be the determinant for the utilization of ANC services. Paternal occupation and religion have been documented as some of the predictors of maternal healthcare utilization among mothers in Harare, Zimbabwe [42]. A systematic review of barriers to accessing maternal care in low‑income countries in Africa found economic factors and cultural beliefs to be barriers to maternal healthcare utilization [27]. Similarly, a survey on utilization of ANC services in sub‑Saharan Africa found that money needed for treatment was the only barrier to having at least eight ANC visits in Nigeria, and that families with better income status are more likely to utilize ANC services [43]. Another survey in a resource‑poor setting found religion and maternal education as significant predictors of women’s place of delivery [4, 44]. Also, educational attainment, occupation, and household wealth have been documented as “markers of economic resources which empower women to take control of their health and facilitate easy access to quality maternal health care” [8]. Similar to a finding of this study is the report for a similar survey in Nigeria that region of residence was significantly associated with facility delivery and that women in northern Nigeria were less likely to deliver in a health facility than those who resided in the southern part of the country [8]. A study in Kenya has also found ethnicity to be one of the factors with strong influences on the timing of the first ANC visit and the type of delivery assistance received by participants [39]. Also, insurance type has been documented as one of the enabling factors associated with health service utilization in Korea [45]. This study’s community‑based design and the scientifically sound sampling methodology enhance the validity of its findings. Additionally, rigorous data quality control measures ensured data accuracy. However, the study’s cross‑sectional design and potential for recall bias constitute notable limitations that should be addressed in future research.

## Conclusions

This study highlights the encouraging levels of awareness and utilization of maternal healthcare services among respondents. However, the delayed registration for antenatal care and the primary motivation for postnatal care being child vaccination underscore the need for targeted interventions. To improve the quality and timing of maternal healthcare service utilization and ultimately reduce maternal and infant mortality rates and promote better health outcomes for women and their children, policymakers and healthcare providers should focus on early antenatal care registration, encouraging postnatal care attendance beyond child immunization and addressing the determinants influencing utilization of maternal healthcare services.

## Data Availability

The datasets generated and/or analyzed during the current study are available from the corresponding author on reasonable request.
